# The Natural History of Medial Meniscal Root Tears: A Biomechanical and Clinical Case Perspective

**DOI:** 10.3389/fbioe.2021.744065

**Published:** 2021-09-23

**Authors:** Edward R. Floyd, Ariel N. Rodriguez, Kari L. Falaas, Gregory B. Carlson, Jorge Chahla, Andrew G. Geeslin, Robert F. LaPrade

**Affiliations:** ^1^ University of North Dakota School of Medicine and Health Sciences/Sanford Orthopedics & Sports Medicine, Fargo, ND, United States; ^2^ Twin Cities Orthopedics, Edina-Crosstown Surgery Center, Minneapolis, MN, United States; ^3^ Georgetown University School of Medicine, Washington, D.C., DC, United States; ^4^ University of Minnesota Medical School, Minneapolis, MN, United States; ^5^ Rush University Medical Center, Midwest Orthopaedics at Rush, Chicago, IL, United States; ^6^ Department of Orthopaedics and Rehabilitation, Larner College of Medicine, University of Vermont, Burlington, VT, United States

**Keywords:** meniscus, meniscus root tear, meniscus root repair, meniscal extrusion, osteoarthritis

## Abstract

Posterior medial meniscus root tears (PMMRTs) make up a relatively notable proportion of all meniscus pathology and have been definitively linked to the progression of osteoarthritis (OA). While known risk factors for development of OA in the knee include abnormal tibial coronal alignment, obesity and female gender, PMMRTs have emerged in recent years as another significant driver of degenerative disease. These injuries lead to an increase in average contact pressure in the medial compartment, along with increases in peak contact pressure and a decrease in contact area relative to the intact state. Loss of the root attachment impairs the function of the entire meniscus and leads to meniscal extrusion, thus impairing the force-dissipating role of the meniscus. Anatomic meniscus root repairs with a transtibial pullout technique have been shown biomechanically to restore mean and peak contact pressures in the medial compartment. However, nonanatomic root repairs have been reported to be ineffective at restoring joint pressures back to normal. Meniscal extrusion is often a consequence of nonanatomic repair and is correlated with progression of OA. In this study, the authors will describe the biomechanical basis of the natural history of medial meniscal root tears and will support the biomechanical studies with a case series including patients that either underwent non-operative treatment (5 patients) or non-anatomic repair of their medial meniscal root tears (6 patients). Using measurements derived from axial MRI, the authors will detail the distance from native root attachment center of the non-anatomic tunnels and discuss the ongoing symptoms of those patients. Imaging and OA progression among patients who were treated nonoperatively before presentation to the authors will be discussed as well. The case series thus presented will illustrate the natural history of meniscal root tears, the consequences of non-anatomic repair, and the findings of symptomatic meniscal extrusion associated with a non-anatomic repair position of the meniscus.

## Introduction

The meniscus plays a central role in protecting the overall health of the knee, and, while underappreciated in previous years, the maintenance of its integrity has emerged as a key factor in joint preservation (Sims et al., 2009; Yusuf et al., 2011; Driban et al., 2016; Foreman et al., 2020; Willinger et al., 2020). The current literature reports that 78% of patients under the age of 60 undergoing total knee arthroplasty (TKA) have sustained medial meniscal root tears (Choi and Park, 2015). However, lack of recognition, under-diagnosis, and neglect of meniscal root tears continue to be a preventable cause of osteoarthritis and TKA (LaPrade et al., 2014; Packer and Rodeo, 2009; Stärke et al., 2010a; Cinque et al., 2018).

A meniscal root tear may be defined as a complete radial tear within 1 cm of the anterior or posterior tibial attachment of the menisci or an avulsion of the attachment (LaPrade et al., 2014; Cinque et al., 2018; LaPrade et al., 2015a). Injuries causing meniscal root tears tend to occur during deep squatting or activities involving flexion, usually occurring concurrently with some type of rotation (LaPrade et al., 2014; Matheny et al., 2015). Injuries contributing to radial tears are often predicated by degenerative changes of the meniscus, which commonly occur in adults in their fourth or fifth decade of life and/or in obese individuals (Hwang et al., 2012; Brophy et al., 2019; Krych et al., 2020).

The association of medial meniscal root tears with progression to osteoarthritis of the knee has been well-documented in clinical studies. The circumferential fibers of the meniscus distribute vertically oriented compressive forces across the knee into axially directed “hoop stresses,” thereby evenly distributing the load to the chondral surfaces (Athanasiou and Sanchez-Adams, 2009; Bansal et al., 2021). Meniscus root tears disrupt this biomechanical function, leading to increases in average contact pressure in the medial compartment (59–78%), secondary to a decrease in contact area (36–37% relative to the intact state) (Padalecki et al., 2014). These increases in average contact pressure may be greater in cases of concurrent varus deformity (Lim et al., 2008). The biomechanical consequences of a complete medial meniscus posterior root tear (MMPRT) are equivalent to those following a total medial meniscectomy (Allaire et al., 2008; Bedi et al., 2010). Root avulsions and radial tears up to 10 mm from the root attachment have been biomechanically validated to be functionally similar, resulting in significantly decreased contact areas at all flexion angles, as well as significantly increased mean and peak contact pressures (Padalecki et al., 2014; LaPrade et al., 2015b). This altered force profile in the knee may lead to meniscal extrusion and subsequent loss of articular cartilage (Athanasiou and Sanchez-Adams, 2009; Bedi et al., 2010).

This article will seek to explore the cause-effect and temporal relationship between meniscus root tears, meniscal extrusion, and development of the hallmarks of OA and its clinical implications. The authors will explain the anatomy and biomechanics for this natural history first, followed by case examples from the senior author’s practice to provide illustration of the degenerative process following medial meniscus posterior root tears.

### Anatomy

The meniscal root attachments play an integral role in anchoring the meniscus and preventing extrusion (LaPrade et al., 2014). Non-anatomical repair of the meniscal roots is reported to lead to osteoarthritic changes and may eventually necessitate TKA; thus, precise anatomic landmarks to guide arthroscopic meniscal root repair have been established (LaPrade et al., 2014; Johannsen et al., 2012a; Krych et al., 2017). The medial meniscus posterior root attachment (MPRA) is 9.6 mm posterior and 0.7 mm lateral to the medial tibial eminence (MTE) apex (or 11.5 ± 0.9 mm in direct distance) (LaPrade et al., 2014). The MPRA is 18.0 mm anterior to the posterior margin of the tibial plateau (James et al., 2014). The MPRA is 3.5 mm lateral to the articular cartilage inflection point of the medial plateau and 8.2 mm from the nearest point of the posterior cruciate ligament (PCL) footprint (Johannsen et al., 2012a). The footprint area of the MPRA is a mean of 30.4 mm^2^. Three of the four meniscal roots, the posteromedial, posterolateral and anteromedial (PM, PL, and AM, respectively) also possess supplementary fibers which contribute significantly to the ultimate failure strength of each root attachment. Notably, the shiny white fibers (SWFs) of the PM root have been reported to account for up to 47.8% of the native root strength, and failure to incorporate these structures may impair the efficacy of current repair techniques (Ellman et al., 2014).

Magnetic resonance imaging (MRI) is the gold standard to diagnose root tears with a specificity of 73% and sensitivity of 77% and NPV of 97% (Lee et al., 2014). A pathognomonic sign of meniscus root tear is the absence of an identifiable meniscus on sagittal sequence (referred to as the “ghost sign”) (LaPrade et al., 2014; Cinque et al., 2018; Chahla et al., 2016). Other characteristic signs of meniscal tear include increased fluid accumulation around the meniscal root, >3 mm meniscal extrusion on a coronal image from the tibial articular edge, and “truncation signs” which are vertical line defects at the meniscal root attachment (James EWJ et al., 2017). Physical exam alone is often ineffective at diagnosing a posterior meniscus root tear, though palpable extrusion can sometimes be detected (Seil et al., 2011). A thorough history of pain with deep flexion, limited activity, and sometimes an popping sensation at the time of the inciting event can be helpful in diagnosing posterior meniscus root tear.

### Biomechanics

*In vitro*, biomechanical studies report that a meniscal root tear is equivalent to a total meniscectomy. Allaire et al. (2008) demonstrated that there was no difference in contact pressures between a meniscal root tear and a complete meniscectomy, suggesting their functional similarity (Allaire et al., 2008). The cadaver study found that a posterior root tear of the medial meniscus resulted in an increase of peak contact pressure of 25% in comparison to an intact meniscus. Furthermore, the authors found a 2.98° increase in external rotation and 0.84 mm increase of lateral tibial translation in knees with root tears. Contact pressure and rotation/translation were restored following repair.

Padalecki et al. examined the changes in average compartment pressures and contact area after medial meniscus root avulsions and radial tears at 3-, 6-, and 9 mm from the root, along with changes found after an anatomic repair. According to the authors’ findings, the contact area for an intact meniscus ranges from 360 mm^2^ at 90° to 450 mm^2^ at 0°. Pooled findings across ROM conditions indicated that the actual contact area of the knee had an average 36% decrease for PMMR avulsions and 37% decrease for complete radial tears at 3-, 6-, and 9 mm from the root attachment. The authors found that average medial compartment contact pressures increased by 69% after root avulsions and 59–78% for radial tears near the root. For knee flexion angles greater than 0°, a meniscal root repair with a transtibial pullout technique restored contact pressures and area close to the normal range to be statistically indistinguishable (Padalecki et al., 2014).

One study reported that a 5 mm posteromedial fixation from the anatomic root attachment site (nonanatomic repair) resulted in increased average contact pressures and decreased contact area when compared to both the intact state and an anatomic repair (Packer and Rodeo, 2009). An anatomic root repair was reported to lead to significantly higher contact area, as well as lower average contact pressures across 0°, 30°, 60°, and 90° of flexion. Peak contact pressures were significantly increased for nonanatomic repairs by 59% relative to the intact state when pooled across all flexion angles, and peak contact pressures at 90° were significantly higher (+47%) for nonanatomic repairs in comparison to anatomic repairs (LaPrade et al., 2015b). These patients present in clinic with classic symptoms of OA, which include progressive pain and swelling.

Meniscal extrusion usually occurs secondary to medial meniscal root tears. As a consequence of extrusion, the torn meniscus may become adherent to the posteromedial joint capsule. Nonanatomic repairs may ultimately fail because they secure the meniscus in this non-functional, extruded position. Meniscal extrusion has been correlated with radiographic signs of osteoarthritis (Strauss et al., 2016). Despite the success of an anatomic repair under ideal circumstances, cyclic loading, representative of postoperative rehabilitation, or suture cut-out from the meniscal tissue, present ongoing problems even with correct surgical technique (LaPrade et al., 2015c). Use of an additional transtibial centralization suture may successfully treat meniscal extrusion and provides an additional point of fixation thereby protecting the repair (Daney et al., 2019; Dean et al., 2020a). Additional considerations include the effects of coronal malalignment; varus alignment has been linked to increased medial meniscus extrusion (Willinger et al., 2020).

Clinically, meniscectomy and non-operative treatment have shown inferior outcomes in preventing the progression of osteoarthritis (Stärke et al., 2010b; LaPrade et al., 2015b; Faucett et al., 2019). It has been reported in one study that, over 10 years, patients with medial meniscus root tears undergoing meniscectomy and nonoperative management progress to osteoarthritis at rates of 99.3 and 95.1%, respectively, in comparison to a significantly lower rate found following meniscus root repair (53.0%) (Faucett et al., 2019). However, the rate of osteoarthritis progression is variable and dependent on patient health and activity level. Another study reported that 87% of menisci treated non-operatively failed, with 31% of cases resulting in eventual total knee arthroplasty (TKA) (Krych et al., 2017). This results in the need for TKA or a subsequent surgery to release the extruded meniscus and return it to an anatomic location (LaPrade et al., 2015b; Choi and Park, 2015; Faucett et al., 2019).

### Transtibial Two-Tunnel Pullout Repair

Indications for repair include healthy, active patients with PMMRTs. While age cutoffs have been sometimes applied, in the senior author’s practice the relative health and activity level of the patient plays a large role in determining their potential benefit from an anatomic root repair. Advanced radiographic signs of OA is a counterindication.

If the meniscus root tear is scarred in an extruded position, an extensive peripheral release using scissor biters is performed first to separate the meniscus from the capsular wall (Dean et al., 2020b). An anatomic two tunnel transtibial pullout root repair is then performed to reposition the meniscus back into an anatomic position. In the transtibial pullout technique, the meniscal root is re-anchored to the tibial plateau by passing sutures through the meniscal root and retrieving them through transtibial tunnels (Dean et al., 2020b).

Initial incisions for the transtibial tunnels are made just medial to the tibial tubercle. The root attachment site is cleared to bleeding bone with a curette to facilitate healing (Dean et al., 2020b). The first tunnel is created along the posterior aspect of the posterior root attachment site using a tibial tunnel guide, while the second tunnel is created approximately 5 mm from the initial tunnel using an offset guide (Dean et al., 2020b). The torn meniscal root is repositioned into its anatomic location. A self-capture passer helps in passing two sutures or suture-tapes in a simple or vertical mattress configuration through the far posterior portion of the detached meniscal root, approximately 5 mm from the medial meniscus’s lateral edge (Dean et al., 2020b). Lee et al. reports that using two sutures for repair showed improved outcomes for patients (Lee et al., 2014). As the sutures are passed through transtibial tunnels, care should be taken to avoid soft tissue bridging and a cannula may be used to facilitate suture shuttling (Dean et al., 2020b). The sutures are secured over a cortical fixation device on the anteromedial tibia with the knee flexed at 90° (Cinque et al., 2017). The posterior root of the medial meniscus should be visualized arthroscopically to confirm a secure repair.

## Cases

### Nonanatomic Repair

From the authors’ practices, six patients who underwent revision root repair for initial nonanatomic posterior medial meniscus root tear (PMMRT) repairs were examined. (Table 1) These patients continued to exhibit painful symptoms and progression of osteoarthritis after a nonanatomic medial meniscus repair. The distance of the initial nonanatomic root repairs was measured on axial MRI, with measurements taken using an electronic image storage system client (IMPAX client 6 AGFA, Mortsel, Belgium) for picture archiving and communication system (PACS). The most proximal axial MRI slice was identified in which the transtibial root repair tunnel from the initial surgery could be seen. A line was then measured from the center of the nonanatomic tunnel to the center of the native PMMR attachment site, as determined by measurements given in Johannsen et al. (2012b).

**TABLE 1:** This table illustrates the distances between the tibial tunnel and the medial meniscus posterior root attachment. These measurements were taken using axial views of the MRIs of patients who underwent nonanatomic repair of the posterior medial meniscal root.

Patients with nonanatomic repairs	Tibial tunnel measured distance from anatomic medial meniscus posterior root attachment (mm)
Patient #1	12.9
Patient #2	8.2
Patient #3	14.0
Patient #4	15.5
Patient #5	11.4
Patient #6	13.5

The distances thus measured ranged from 8.2 to 14.0 mm, representing the distance from the initial tunnel to the anatomic attachment area (Table 1). Extrusion of the medial meniscus was noted in all cases. The interval of these patients from initial evaluation to revision ranged 5–17 months (date of initial surgery only available for patients 1, 2, 4 and 6). Patients were predominantly in their fifth decade.

**FIGURE 1:**
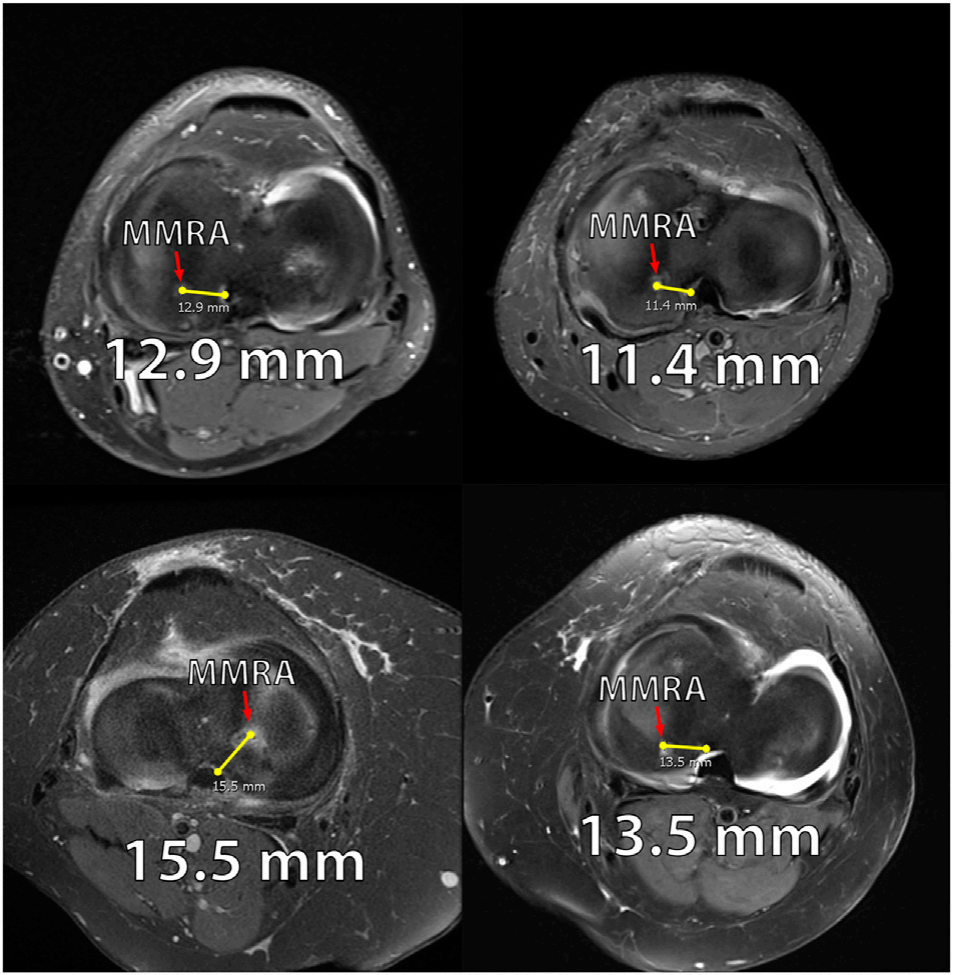
Axial MRIs of 4 patients from the nonanatomic meniscal root reconstruction cohort. **(A)**: MRI of Nonanatomic Patient #1 displaying the medial meniscus root attachment 12.9 mm from anatomic attachment site (right knee). **(B)**: MRI of Nonanatomic Patient #5 displaying the medial meniscus root attachment 11.4 mm from anatomic attachment site (right knee). **(C)**: MRI of Nonanatomic Patient #4 displaying the medial meniscus root attachment 15.5 mm from anatomic attachment site (left knee). **(D)**. MRI of Nonanatomic Patient #6 displaying the medial meniscus root attachment 13.5 mm from anatomic attachment site (right knee).

**FIGURE 2:**
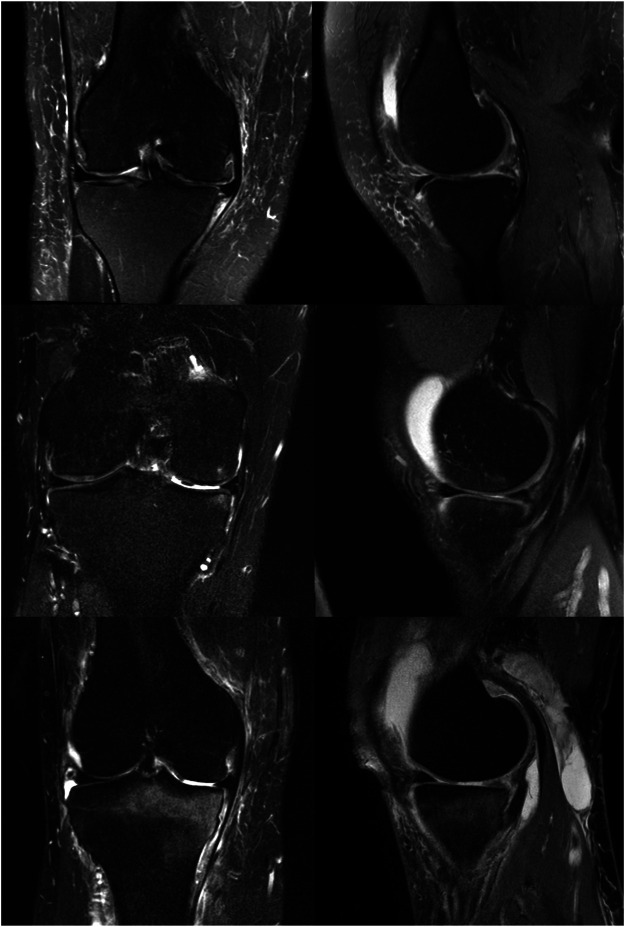
Coronal and Sagittal MRIs of patients from the natural history cohort. **(A)**: Coronal and sagittal MRI of Natural History patient #1 illustrating medial meniscus extrusion and mild bony edema (right knee). **(B)**: Coronal and sagittal MRI of Natural History patient #3 illustrating mild medial meniscus extrusion and moderate bony edema (right knee). **(C)**: Coronal and sagittal MRI of Natural History Patient #6 illustrating mild medial meniscus extrusion and moderate to severe bony edema (right knee).

**FIGURE 3:**
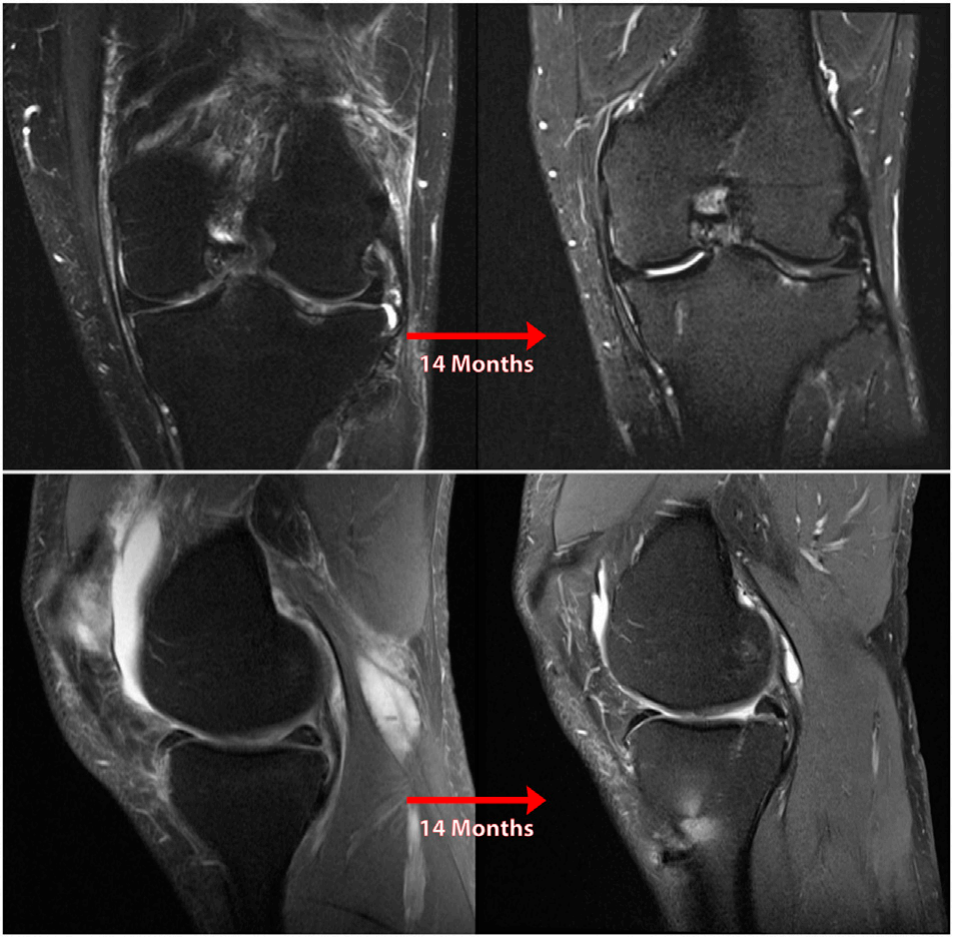
Coronal **(A)** and sagittal **(B)** MR images of nonanatomic root repair patient #1 showing progression of osteoarthritis in the medial compartment of the knee over 14 months secondary to nonanatomic medial meniscus root repair (left knee). M = medial; L = lateral; A = anterior; P = Posterior.

**FIGURE 4:**
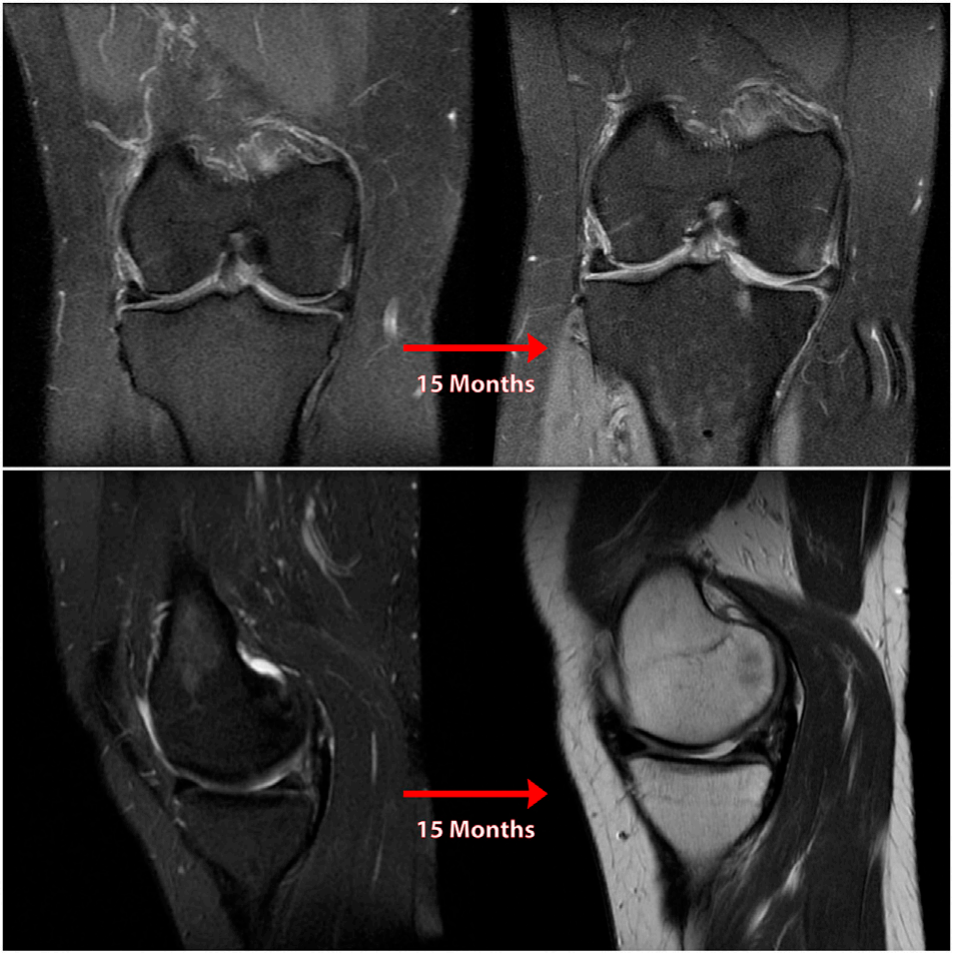
Coronal **(A)** and sagittal **(B)** MR images of nonanatomic root repair patient #2 showing progression of osteoarthritis in the medial compartment of the knee over 15 months secondary to nonanatomic medial meniscus root repair (right knee). M = medial; L = lateral; A = anterior; P = Posterior.

**FIGURE 5:**
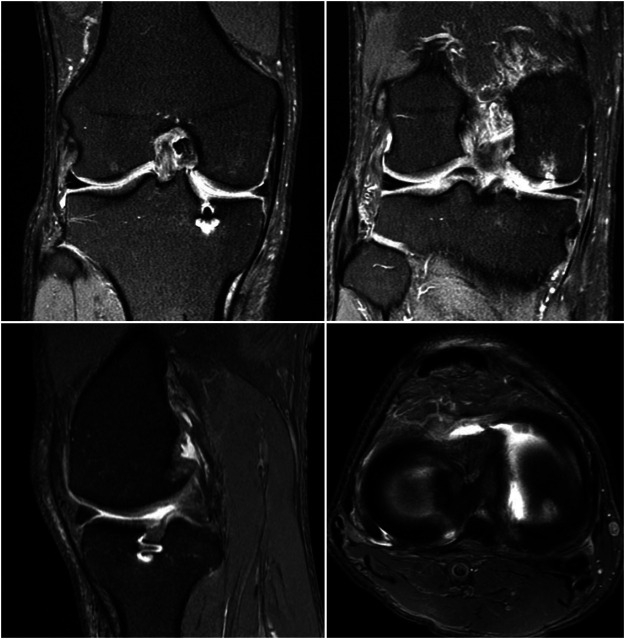
Coronal **(A and B)**, sagittal **(B)**, and axial **(C)** MR images of nonanatomic root repair patient #3 showing evidence of osteoarthritis in the medial compartment of the knee secondary to nonanatomic medial meniscus root repair (right knee). M = medial; L = lateral; A = anterior; P = Posterior.

**FIGURE 6:**
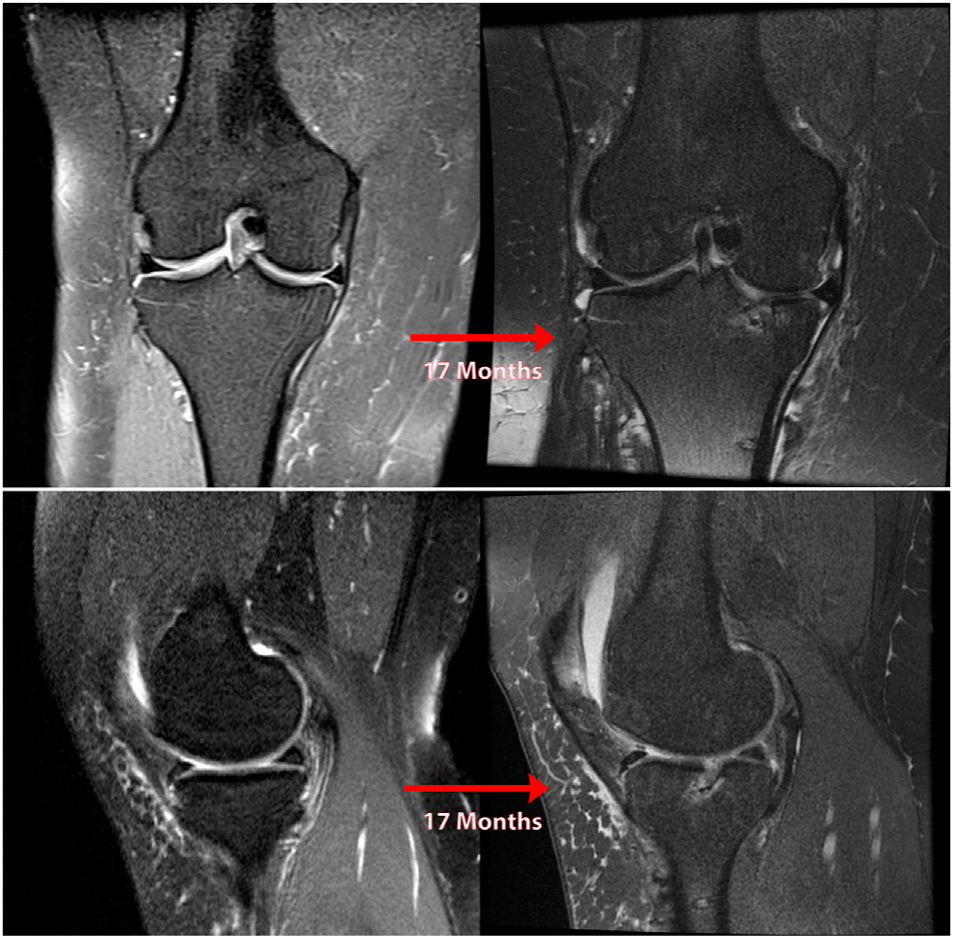
Coronal **(A)** and sagittal **(B)** MR images of nonanatomic root repair patient #4 showing progression of osteoarthritis in the medial compartment of the knee over 17 months secondary to nonanatomic medial meniscus root repair (right knee). M = medial; L = lateral; A = anterior; P = Posterior.

**FIGURE 7:**
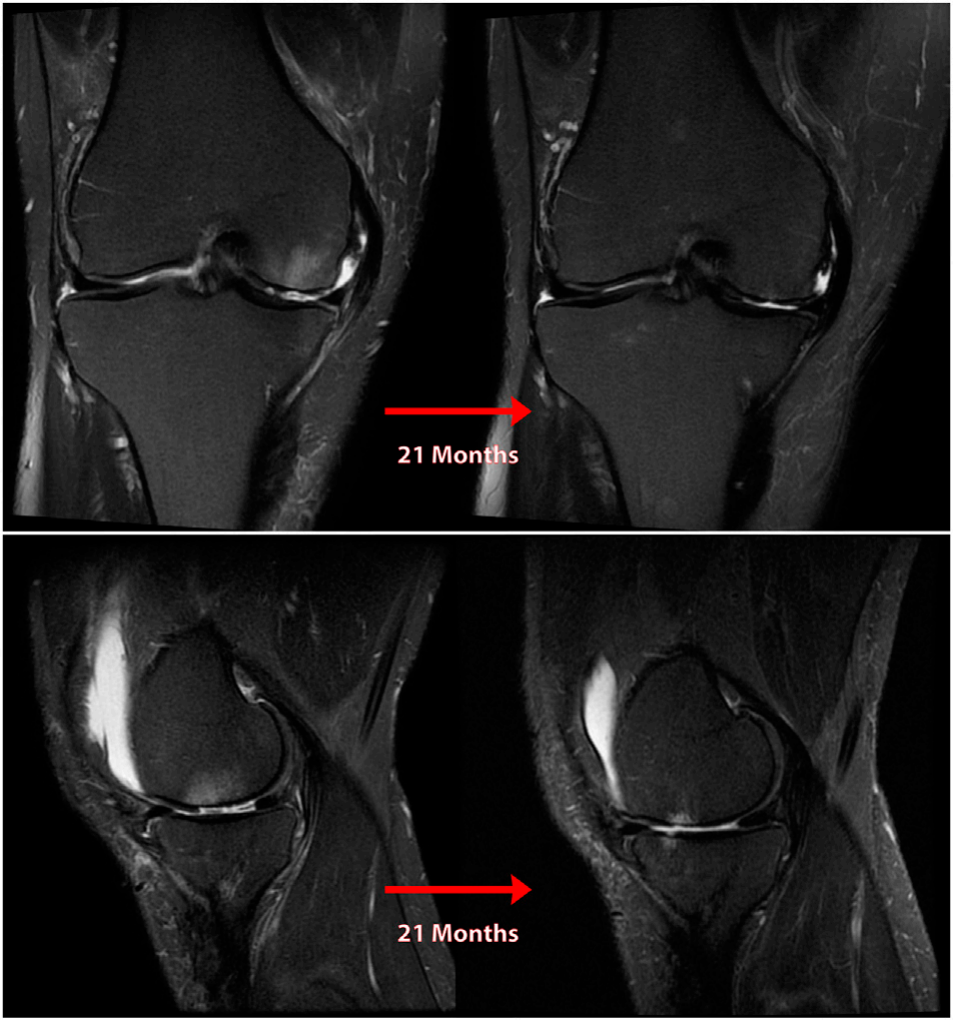
Coronal **(A)** and sagittal **(B)** MR images of nonanatomic root repair patient #5 showing progression of osteoarthritis in the medial compartment of the knee over 21 months secondary to nonanatomic medial meniscus root repair (right knee). M = medial; L = lateral; A = anterior; P = Posterior.

**FIGURE 8:**
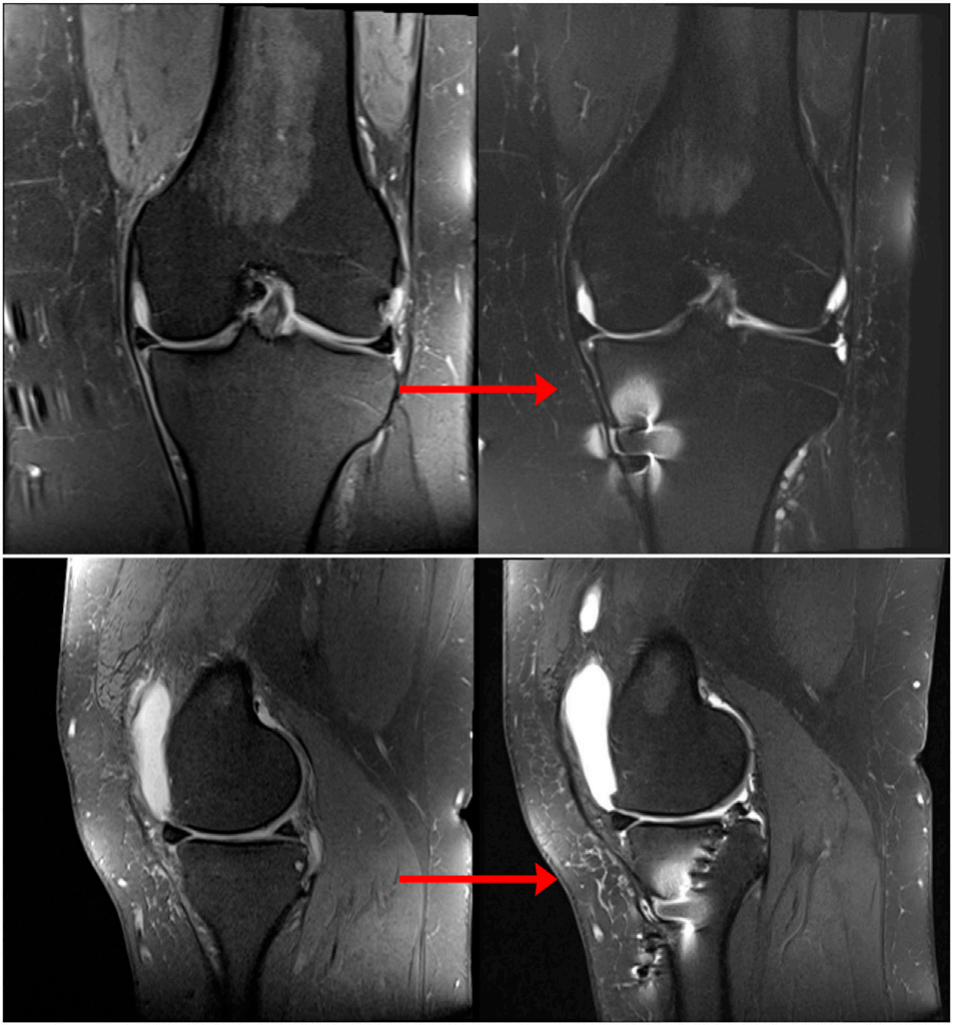
Coronal **(A)** and sagittal **(B)** MR images of nonanatomic root repair patient #6 showing progression of osteoarthritis in the medial compartment of the knee over 12 months secondary to nonanatomic medial meniscus root repair (left knee). M = medial; L = lateral; A = anterior; P = Posterior.

For these patients, revision meniscus root repair was indicated. The extruded meniscus was released from the capsule, and an anatomic meniscus root repair was performed in the manner outlined above. Follow-up reports at 1 year were available for patients 1, 2 and 4. They reported improvement in symptoms and radiographic signs of osteoarthritis showed no progression. (See Figures 1–8).

### Progression of Osteoarthritis in Untreated Meniscus Root Deficiency

Another five patients were examined who had identifiable PMMRTs at some point in the past and did not undergo meniscus root repair. Subsequent imaging demonstrated the progression of osteoarthritis (OA) following their untreated tear, following the known natural history of posterior meniscus root lesions. Information regarding these patients, in whom the natural history of OA progression can be observed following meniscus root tear, is given in Table 2.

**TABLE 2:** This table illustrates characteristics and timelines of the natural history of medial meniscus root tears. Cartilage degradation characteristics and extent of meniscal extrusion can be seen for each patient who had a neglected posterior medial meniscus root tear.

Natural history patients	Femorotibial joint space narrowing	Interval from injury (months)	Cartilage defect size	Cartilage defect grade	Meniscus extrusion
Natural History #1	Not applicable	60	Not applicable	Not applicable	Not applicable (unicompartmental arthroplasty)
Natural History #2	None	6	Not Measured	4	3 mm
Natural History #3	Not applicable	24	Not applicable	Not applicable	Not Applicable
Natural History #4	None	5	0.9 × 1.7 cm	4	3 mm
Natural History #5	None	15	Not measured	2	5 mm

Imaging studies verifying PMMRTs was available for each of these patients, whether from a prior provider or from the senior author’s practice. The presence of a complete medial meniscus root tear without end-stage OA was verifiable on prior imaging for most of these patients, and new MRIs were obtained demonstrating the development of chondromalacia, full-thickness cartilage defects, meniscal extrusion, and joint space narrowing. Each of these patients is detailed below.

**FIGURE 9:**
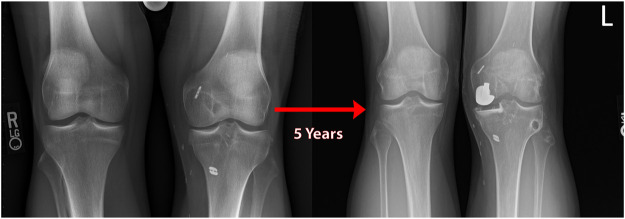
Standing bilateral radiographs of the knee of natural history patient #1 showing results of a unilateral knee arthroplasty used to treat medial compartment osteoarthritis over 5 years following an iatrogenic medial meniscal root tear (left knee).

Patient 1, a 30-year-old male, suffered an iatrogenic PMMRT during posterior cruciate ligament (PCL) reconstruction, at which time the cartilage surfaces appeared healthy. The meniscus root was not repaired, and 5 years later severe medial compartment OA necessitated unicompartmental knee arthroplasty at the age of 29. The patient’s course of treatment was complicated by an initially untreated PMMRT. (See Figure 9).

Patient 2, a 39-year-old male, suffered a PMMRT and was treated with partial medial meniscectomy (PMM). Within 6 months, the patient was highly symptomatic and an MRI at the authors’ practice demonstrated grade III-IV chondromalacia of the MFC and medial tibial plateau (MTP) over a 15 × 18 mm area on both surfaces, in addition to a full-thickness radial tear a the far posterior PHMM.

**FIGURE 10:**
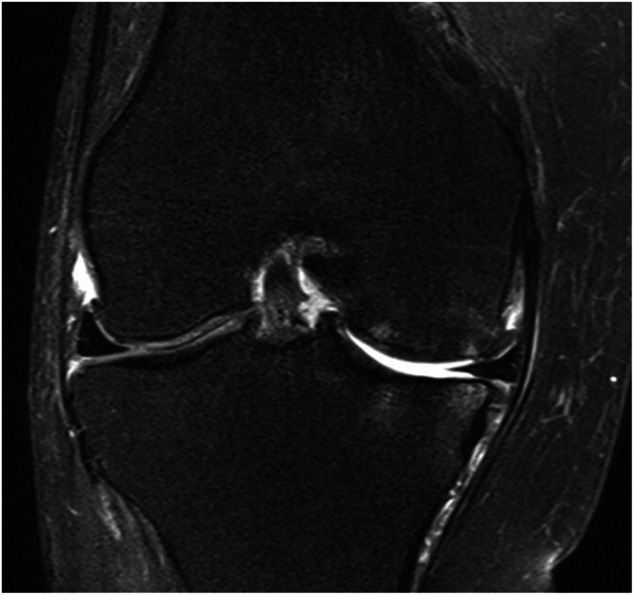
Coronal MRI of the knee of natural history patient #3 showing progression of osteoarthritis over 5 months following neglected medial meniscal root tear (right knee).

Patient 3, a 43-year-old female, experienced a PMMRT and was seen in the senior author’s practice. An MRI at the time of initial visit demonstrated normal, healthy cartilage surfaces. The patient declined treatment, and at 6 months after the initial visit, communicated that she was increasingly symptomatic with pain and functional limitation. Returning at 2 years after injury, new MRIs were obtained (See Figure 10) which demonstrated meniscal extrusion, abundant subchondral edema, loss of cartilage surface on the MFC and MTP weight-bearing areas and decreased medial joint space on standing Rosenberg radiographs.

**FIGURE 11:**
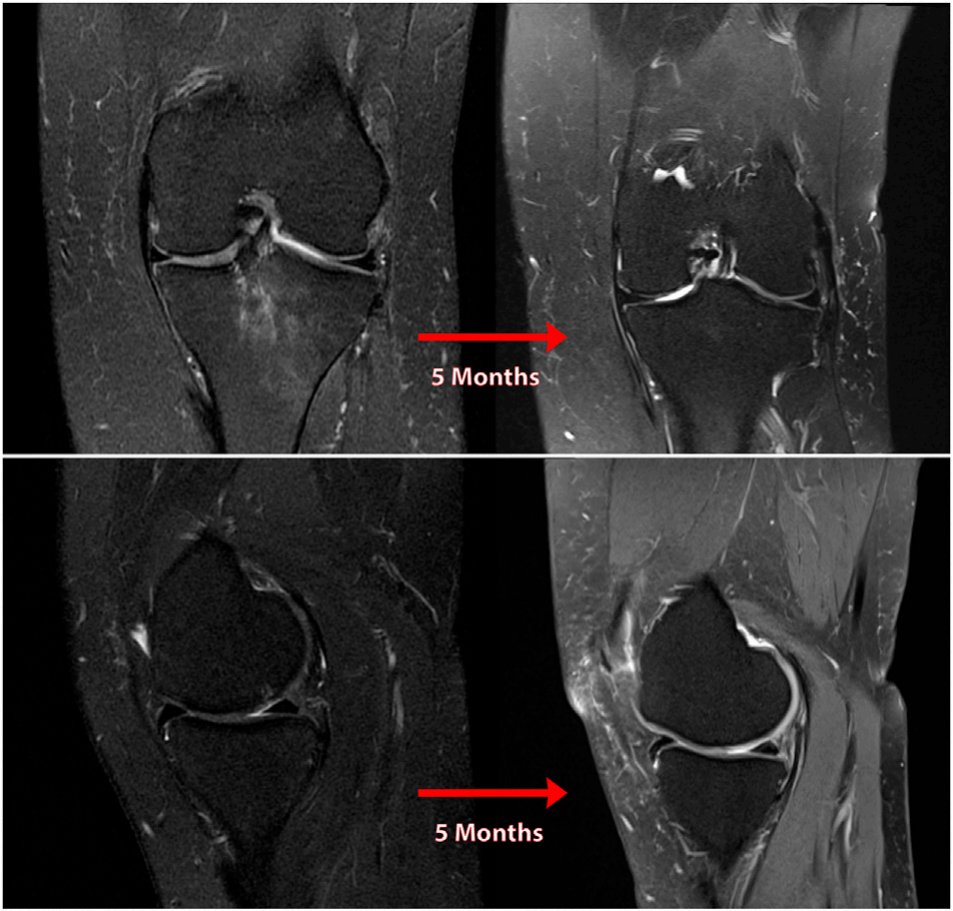
Coronal **(A)** and sagittal **(B)** MR images of the knee of natural history patient #4 showing progression of osteoarthritis over 5 months following neglected medial meniscal root tear (left knee).

Patient 4, a 57-year-old female, presented/experience pain for 5 months that began after going for a walk, radiographs and MRI showed preserved joint space and cartilage at the 4-month point. The patient’s MRI at her office visit, however, revealed a full-thickness radial tear through the PHMM/root junction along with 3 mm of medial meniscus extrusion. Diffuse full-thickness chondral fissuring and signs of OA were noted on the weight-bearing surfaces of both the MTP and lateral femoral condyle (LFC). (See Figure 11).

**FIGURE 12:**
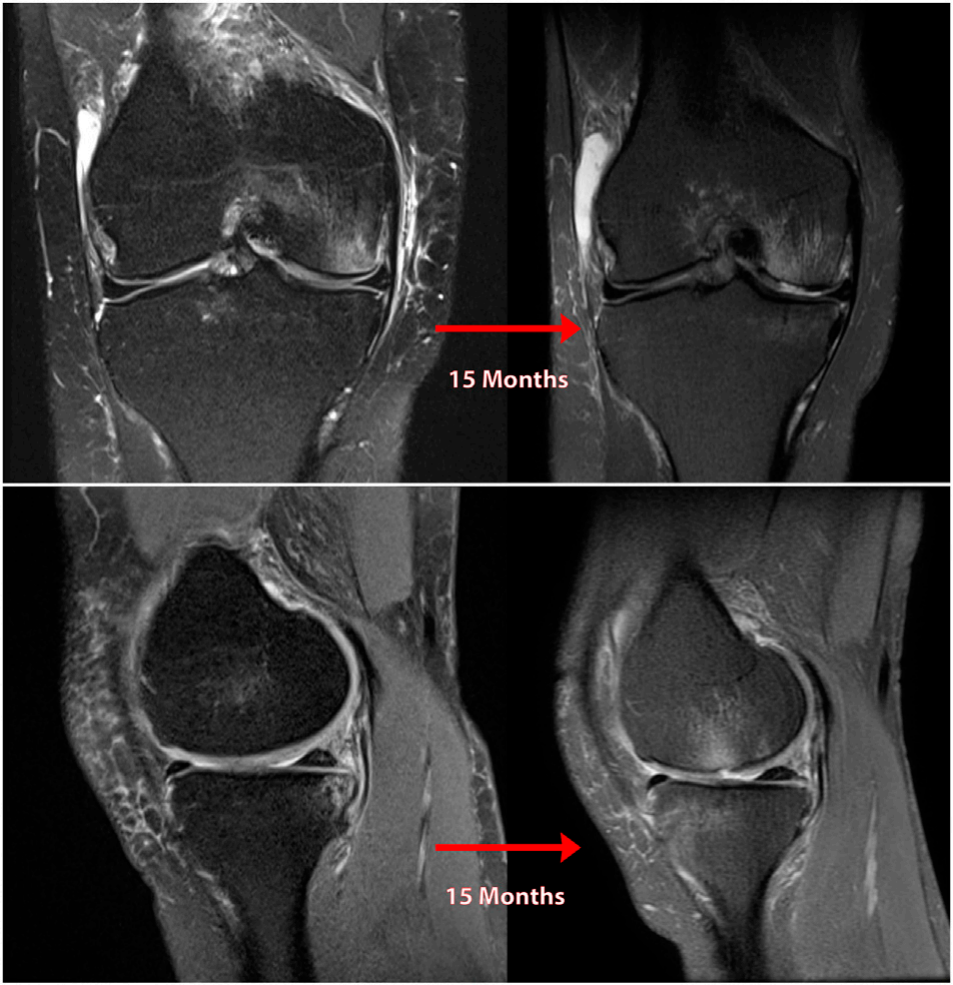
Coronal **(A)** and sagittal **(B)** MR images of the knee of natural history patient #5 showing progression of osteoarthritis over 5 months following neglected medial meniscal root tear (right knee).

Patient 5, a 49-year-old female, suffered a PMMRT after a biking injury. She was evaluated 4 weeks later and found to a medial meniscal root tear with some extrusion, along with mild chondromalacia of the medial compartment. On subsequent evaluation 15 months later, an MRI demonstrated grade II chondromalacia on the medial tibial plateau, patellofemoral and medial compartment OA, 5 mm of meniscal extrusion and a marked joint effusion in the absence of any ligamentous deficiency. Clinical evaluation revealed no other probable cause for this rapid progression to OA. (See Figure 12).

## Discussion

**FIGURE 13:**
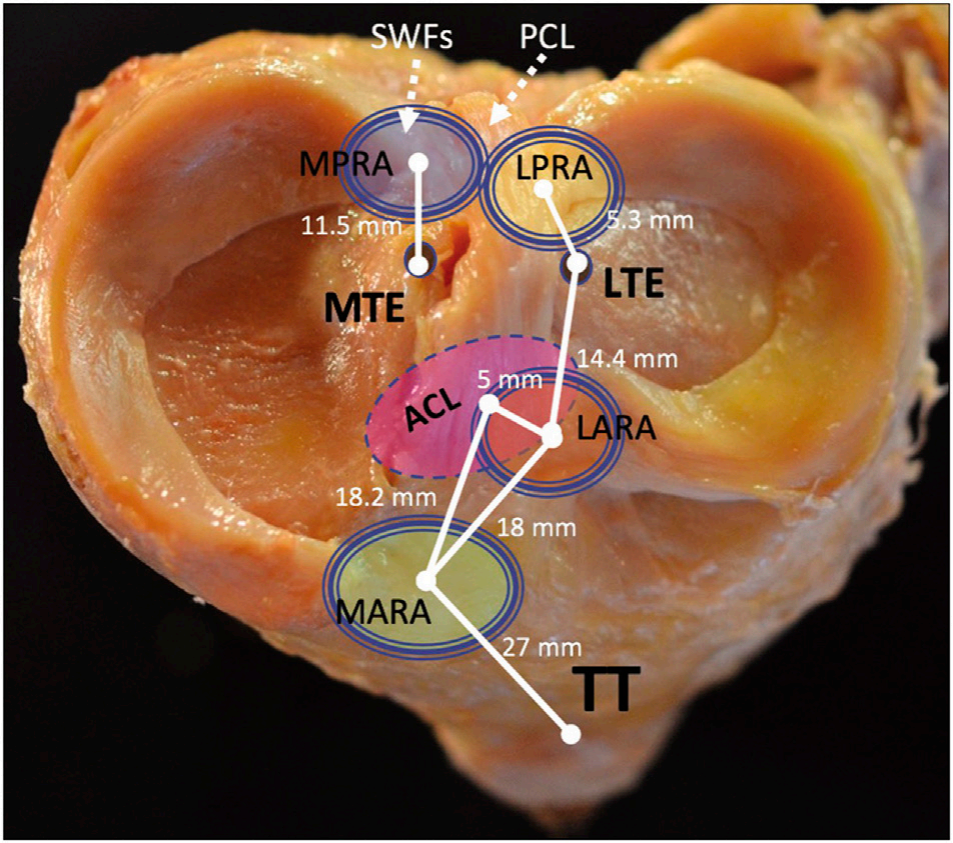
The tibial plateau, medial and lateral menisci, and important points of arthroscopic anatomy. MPRA, medial posterior root attachment; LPRA, lateral posterior root attachment; LARA, lateral anterior root attachment; MARA, medial anterior root attachment; ACL, anterior cruciate ligament; TT, tibial tubercle; MTE, medial tibial eminence; LTE, lateral tibial eminence. Figure first appeared in: “LaPrade RF, Floyd ER, Carlson GB, Moatshe G, Chahla J, Monson JK. Meniscal Root Tears: Solving the Silent Epidemic. J Arthrosc Surg Sports Med 2021; 2 (1):47–57.”

Appreciation of the deleterious effects of meniscal root tears has grown in recent years, as has knowledge of the biomechanical role of the meniscus in the knee. While in the past, common practice involved complete or partial meniscectomy in treatment of many meniscal tears, advances in understanding of meniscal anatomy and of the importance of meniscal integrity in prevention of OA have led to increasing preference for repair over resection (8) (Figure 13). Meniscus root tears, in particular, have occupied a somewhat peculiar space, being underdiagnosed and often neglected.

However, biomechanical studies have demonstrated the clear link between posterior meniscal root tears and the progression of OA (Stärke et al., 2010b; Bansal et al., 2021). Long term high-level studies of meniscal root repair are still forthcoming; however, it is the authors’ contention that biomechanical work validating the natural history of PMMRTs as equivalent to meniscectomy provides sufficient evidence to warrant repair in these injuries. In this study, we have presented a case series of patients who underwent nonanatomic PMMRT repairs and patients who presented with OA as a result of neglected medial meniscal root tears. In the author’s practice, MRI is always obtained to evaluate if the meniscus is repairable. If the meniscus is repairable and the patient has a favorable profile for repair, the senior author will repair the meniscal root regardless of if it has a degenerative or traumatic etiology. All patients demonstrated a progression of medial compartment knee arthritis as predicted by previous biomechanical studies.

From a clinical standpoint, the observations made from biomechanical studies are borne out by the relatively fast development of OA after PMMRT, sometimes in a matter of months in the patients observed by these authors. As seen in this case series, unnecessary meniscectomy continues to be a significant driver of the development of OA in orthopedic practices all over the US. A case series of nonanatomic repairs of posterior medial meniscus root tears has not been presented previously, and these cases suggest that such repairs can reliably lead to a continuation or worsening of symptoms, and often require revision surgery. Meniscal extrusion after PMMRTs may partially explain the number of nonanatomic repairs seen nationwide; the meniscus often heals with scar tissue in the extruded position adjacent to the capsule and without some form of arthroscopic release is not readily reducible to anatomic position (DePhillipo et al., 2019). Biomechanical studies have given rationale, observed in the failure of the non-anatomic repairs presented here, that root repair which fail restore the anatomical position of the meniscus attachment are equivalent to meniscectomy (Stärke et al., 2010a; Padalecki et al., 2014; LaPrade et al., 2015b). Increased contact pressure in biomechanical studies may be correlated to progression of OA in untreated PMMRTs.

As noted above, indications for PMMRT repair can be dependent on the patient’s age and activity status. Radiographic absence of arthritis and active lifestyle should be counterbalanced against patients’ age alone. Younger patients without significant cartilage defects or joint space narrowing and MRI evidence of meniscal root tear should be considered good candidates for this surgery.

Limitations of this report include complex histories and not examining the potential relation between patient weight, alignment, and progression of symptoms. For some patients who presented without a clear inciting event, the actual duration of time may be uncertain in which the weight-bearing cartilage surfaces were exposed to increased contact forces due to PMMRT. An additional limitation may be selection bias in presenting primarily patients from a private sports medicine practice in the midwestern United States. However, the senior author’s practice is a tertiary referral practice which serves patients from across and outside of the United States; therefore, any selection bias may be more socioeconomic than geographical.

## Conclusion

Root tears of the posterior medial meniscus can be potentially debilitating injuries, leading to pain, limitation of activity, and decreased quality of life. Biomechanical studies have suggested an additional link between PMMRTs and the progression of OA, and a high incidence of untreated PMMRTs has been reported among younger patients undergoing TKA. Anatomic reduction of the meniscus root attachment and restoration of the native root position restores the integrity of the knee and prevents progression of this natural history. A heightened awareness for this injury pattern is necessary with prompt MRI if history and exam suggest a possible meniscal root tear. Magnetic resonance imaging requires careful scrutiny by surgeons to ensure identification of these tears. At the time of surgery, specific attention to the posterior meniscal roots and including probing the structures at the time of knee arthroscopy is recommended. In appropriately selected patients, repair of a meniscus root tear should be performed with a validated repair construct (i.e., two sutures secured through transtibial tunnels) with consideration of a peripheral release to anatomically reduce the meniscus.

## Data Availability

The original contributions presented in the study are included in the article/Supplementary Material, further inquiries can be directed to the corresponding author.
